# Genome-wide association reveals three SNPs associated with sporadic amyotrophic lateral sclerosis through a two-locus analysis

**DOI:** 10.1186/1471-2350-10-86

**Published:** 2009-09-09

**Authors:** Qiuying Sha, Zhaogong Zhang, Jennifer C Schymick, Bryan J Traynor, Shuanglin Zhang

**Affiliations:** 1 Department of Mathematical Sciences, Michigan Technological University, Houghton, MI, USA; 2 School of Computer Science and Technology, Heilongjiang University, Harbin, PR China; 3 Laboratory of Neurogenetics, National Institute on Aging, NIH, Bethesda, MD, USA; 4 Department of Physiology, Anatomy and Genetics, University of Oxford, Oxford, UK; 5 Department of Neurology, Johns Hopkins University, Baltimore, MD, USA; 6 Department of Mathematics, Heilongjiang University, Harbin, PR China

## Abstract

**Background:**

Amyotrophic lateral sclerosis (ALS) is a fatal, degenerative neuromuscular disease characterized by a progressive loss of voluntary motor activity. About 95% of ALS patients are in "sporadic form"-meaning their disease is not associated with a family history of the disease. To date, the genetic factors of the sporadic form of ALS are poorly understood.

**Methods:**

We proposed a two-stage approach based on seventeen biological plausible models to search for two-locus combinations that have significant joint effects to the disease in a genome-wide association study (GWAS). We used a two-stage strategy to reduce the computational burden associated with performing an exhaustive two-locus search across the genome. In the first stage, all SNPs were screened using a single-marker test. In the second stage, all pairs made from the 1000 SNPs with the lowest p-values from the first stage were evaluated under each of the 17 two-locus models.

**Results:**

we performed the two-stage approach on a GWAS data set of sporadic ALS from the SNP Database at the NINDS Human Genetics Resource Center DNA and Cell Line Repository http://ccr.coriell.org/ninds/. Our two-locus analysis showed that two two-locus combinations--rs4363506 (SNP1) and rs3733242 (SNP2), and rs4363506 and rs16984239 (SNP3) -- were significantly associated with sporadic ALS. After adjusting for multiple tests and multiple models, the combination of SNP1 and SNP2 had a p-value of 0.032 under the Dom∩Dom epistatic model; SNP1 and SNP3 had a p-value of 0.042 under the Dom × Dom multiplicative model.

**Conclusion:**

The proposed two-stage analytical method can be used to search for joint effects of genes in GWAS. The two-stage strategy decreased the computational time and the multiple testing burdens associated with GWAS. We have also observed that the loci identified by our two-stage strategy can not be detected by single-locus tests.

## Background

Amyotrophic lateral sclerosis (ALS) is a fatal progressive neurodegenerative disease that attacks nerve cells in the brain and spinal cord resulting in muscle weakness and atrophy. Although ALS is listed as a rare disease with a prevalence of approximately 1 per 10,000, it is the most common adult onset form of motor neuron diseases [1,2]. Epidemiological studies have showed that 1.5-5.3% of cases are familial in nature [3-6]. The remaining 95% of cases are not associated with a family history of the disease and seem to occur sporadically throughout the community. Several genes that cause familial ALS have been identified [7-14], especially the SOD1 gene which is believed to be responsible for 20% of familial ALS.

The identification of susceptibility genes of sporadic ALS has been slow in arriving. The search for sporadic ALS genes has generated a large number of candidate-gene association studies [15-19]. To date, we do not have a functional SNP or haplotype that has made a credible contribution to our understanding of disease pathogenesis in the way that the *APOE*-e4 allele does in Alzheimer disease (AD) and the H1 MAPT haplotype does in parkinsonian syndromes [20]. There is an urgent need to understand the genetic architecture of sporadic ALS and ultimately to develop novel drugs for this fatal disease. Sporadic ALS is hypothesized to be a complex disorder in which the disease is modulated by variations in multiple genetic loci interacting with each other and environmental exposures [18]. The lack of major genes may be a reason for the unsuccessful candidate gene studies which investigated one gene at a time.

Recently, Schymick et al. made the first attempt to identify genetic factors that might be relevant in the pathogenesis of sporadic ALS by using a well-designed GWAS [1]. The first stage single-marker analysis performed by Schymick et al. showed that 34 SNPs had a p-value less than 0.0001 with the smallest one being 6.8 × 10^-7^. After adjusted by permutation procedure, none of these SNPs reached the significance level of 0.05. This finding suggests that the ALS phenotype is not driven by a single powerful locus. By testing one marker at a time, the first stage analysis made the implicit assumption that susceptibility loci can be identified through their independent, marginal contributions to the trait variability. More recently, other GWAS in ALS have been conducted by different research groups [21-24]. However, all these GWAS used single-marker analysis. Recent human and animal studies of complex diseases have identified susceptibility genes that marginally contribute to a common trait, to a minor extent only or not at all, but that interact significantly in combined analyses [25-32]. Thus, methods that can account for joint effects of genes may be appropriate for analyzing genome-wide association data sets.

In this article, we used seventeen two-locus models to analyze the previously published genome-wide association data for ALS. We found that three SNPs were significantly associated with sporadic ALS. After we observed the significant two-locus combinations, we further estimated the impact (relative risk and odds ratio) of each of the two-locus combinations on sporadic ALS. It has been recognized that the traditional method will over estimate the odds ratio or relative risk in GWAS [32,33]. Recently, Zollner and Pritchard proposed a new method to estimate penetrance and then odds ratio and relative risk [32]. Through extensive simulation studies, Zollner and Pritchard showed that the estimations of odds ratio and relative risk by their method were not upward biased. By modifying Zollner and Pritchard's method, we proposed a new method to estimate two-locus penetrance, and then estimate the odds ratio, relative risk and sample size needed to replicate the findings for this rare disease.

## Methods

In this section, we will give details of the data set and describe a new analytical method to analyze this data set.

### The Data Set from GWAS for Sporadic ALS

Schymick et al. have made their data set publicly available through the website of the National Institute of Neurological Disorders and Stroke (NINDS) Human Genetics Resource Center at the Coriell Institute http://ccr.coriell.org/ninds[1]. The data set contained 555,352 unique SNPs across the genome in 276 patients with sporadic ALS and 271 neurologically normal controls. The 555,352 SNPs were carefully chosen tagging SNPs from phase I and II of the HapMap Project. The sampled individuals were all non-Hispanic white Americans. There were 102 females and 174 males in cases, and 142 females and 129 males in controls. All sampled individuals had a more than 95% genotype call rate. The average call rate across all samples was 99.6%. Of the 555,352 SNPs studied, the genotype call rate was greater than 99% for 514,088 (representing 92.6% of all SNPs assayed) and greater than 95% for 549,062 (98.9%) SNPs. The phenotype file of this data set contained the status of sporadic ALS, age of onset, site of onset (bulbar-onset, upper-limb-onset, and lower-limb-onset), gender, and smoking status among other information.

### Statistical Analysis

#### Two-locus Analysis Based on Seventeen Two-locus Models

**Figure 1:**
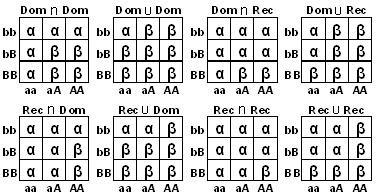
**Eight two-locus epistatic models**. A and B are the high-risk alleles in the two markers. α and β are the penetrance. ∩: two-locus genotypes with both high-risk genotypes at SNP A and SNP B are high-risk genotypes. ∪: two-locus genotypes with at least one high risk genotype at SNP A or SNP B are high-risk genotypes.

**Figure 2:**
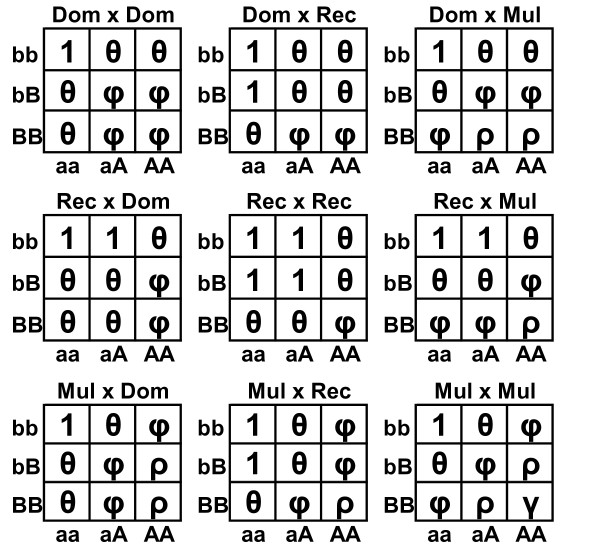
**Nine two-locus multiplicative models**. A and B are the high-risk alleles in the two markers. The symbol in each cell denotes the relative risk of this cell. φ = θ^2^, ρ = θ^3 ^and γ = θ^4^.

In this article, we used seventeen two-locus models to analyze the genome-wide association data. For each SNP, we called one allele a high-risk allele if its frequency in cases was larger than the frequency in controls. For SNP A with alleles A, a and SNP B with alleles B, b, Figure 1 and 2 give eight epistatic two-locus models and nine multiplicative two-locus models with high-risk alleles A and B, respectively. Some of the eight epistatic two-locus models have been used and discussed by Xiong et al. and Zhao et al. [34,35]. The multiplicative models that are good approximations of additive models have been discussed by Hodge and Risch [36,37].

Under each of the epistatic models, the nine two-locus genotypes were divided into two groups: high-risk genotype group and low-risk genotype group. For example, under the model Dom∩Dom, the high-risk group was *G*_*H *_= {*aAbB*, *AAbB*, *aABB*, *AABB*} and the low-risk group was *G*_*L *_= {*aabb*, *aAbb*, *AAbb*, *aaBB*} For the eight epistatic models, we used one degree of freedom (df) *χ*^2 ^test statistic given by

to test for association of two-locus joint effects, where , , and  denote the frequencies of the high-risk genotype group in cases, controls and the pooled sample (cases and controls are pooled together).

For the nine multiplicative models, we constructed a two-locus association test as follows. Let P(*Disease|*g) denote the penetrance of two-locus genotype combination *g *= (*g*_1_, *g*_2_), where *g*_1 _and *g*_2 _are the genotypes in the first and second markers, respectively. Let *β*_0 _denote the logarithm of the penetrance of genotypes with a relative risk of 1 in the models (see Figure 2) and *β*_1 _= log*θ*, where *θ *is the relative risk given in Figure 2. Then, the nine multiplicative models can be described by the following log linear model log *P*(*Disease*|*g*) = *β*_0 _+ *β*_1_*X*, where *X *= *x*_1 _+ *x*_2_, *x*_1 _is the numerical code of *g*_1 _and is given by

for a dominant, recessive or multiplicative model, respectively; *x*_2 _is similarly defined as the numerical code of *g*_2_. Under the log linear model log *P*(*Disease*|*g*) = *β*_0 _+ *β*_1_*X*, *β*_1 _= 0 means that all the genotypes have the same penetrance which implies that *θ *= 1. So a test of the association between the disease and the two loci under the nine multiplicative models is equivalent to a test of the null hypothesis *H*_0_: *β*_1 _= 0. For the *i*^*th *^individual, let *y*_*i *_denote the trait value (1 for diseased individual and 0 for normal individual) and *X*_*i *_denote the numerical code of the genotype (*X *in the log linear model). The score test statistic is given by

where N is the sample size,  is the average of *X*_1_,..., *X*_*N*_, and  is the average of *y*_1_,..., *y*_*N*_. Under the null hypothesis, *T*_*score *_follows a *χ*^2 ^distribution with 1 df. Note that under each of the two-locus epistatic models, if we code *X *= 1 for a high-risk genotype group and *X *= 0 for a low-risk genotype group, then *T*_*epi *_= *T*_*score*_.

The method to search for significant two-locus combinations for each of the seventeen models has the following two steps:

***Step 1***: For each SNP, let *n *and *m *denote the number of individuals in cases and controls (different SNPs may have a different number of cases and controls due to missing genotypes). Let *n*_1_, *n*_2_, *n*_3 _and *m*_1_, *m*_2_, *m*_3 _denote the number of three genotypes in cases and controls, respectively. The 2 df genotypic test statistic is given by

where  and . We applied this test statistic to each SNP, calculated the corresponding p-value, and returned *M *SNPs with the smallest p-values (*M *= 1,000 was used in this article).

***Step 2*: **Under each of the seventeen two-locus models, we applied a two-locus association test to each of the *L *two-locus combinations among the *M *retained SNPs, where *L *= *M*(*M*-1)/2. For a two-locus epistatic model given in figure 1, we used the two-locus test *T_epi_*. For a multiplicative model given in figure 2, we used the score test *T_score_*. In this step, we got a p-value (called raw p-value) for each of the *L *two-locus combinations and each of the seventeen two-locus models.

A permutation procedure was used to adjust for multiple tests and multiple models. In each permutation, we randomly shuffled the cases and controls and repeated step 1 and step 2 based on the permuted data. We performed the permutation procedure *B *times (*B *= 1,000 was used in this article). For the *i*^*th *^model and *l*^*th *^two-locus combination (*i *= 1,...,17; *l *= 1,..., *L*), let *p*_il _and  denote the raw p-values of the two-locus tests in step 2 based on the original data and on the *b*^*th *^permutated data, respectively. Let

Then, for the *i*^*th *^model and *l*^*th *^two-locus combination, P_*il*_, the p-value adjusted for multiple tests and multiple models, was given by .

#### A New Method to Estimate Penetrance

When a study identifies a locus or locus-combination that shows evidence of association with a disease, it is common to estimate the impact of this locus or locus-combination on the phenotype of interest. This impact is often expressed as an odds ratio. Estimation of the odds ratio is also helpful for planning successful replication studies.

It is recognized that the traditional estimate of odds ratio is up-biased because it is typically estimated for the locus which was significant for association [32,33]. Recently, Zollner and Pritchard proposed a new method to estimate penetrance (odds ratio can be calculated based on the penetrance) [32]. This new method was based on the likelihood of observed genotypes given that the locus was significant for association. We modified Zollner and Pritchard's method to estimate the penetrance and odds ratio for two-locus combinations under each of the seventeen models given in Figure 1 and Figure 2. We use the Dom∩Dom model given in Figure 1 as an example to describe our method.

We use the following notation:

*n*, *m*: the number of cases and controls

the data *D *= {*n*_1_,..., *n*_9_; *m*_1_,..., *m*_9_}: the counts of nine two-locus genotypes in cases and controls that constitute the significant signal for association

(*q*_1_,..., *q*_9_): the population frequencies of the genotypes

*R*: the relative risk of high-risk genotype combination to low-risk genotype combination, *R *= *β/α*.

*F*: the population prevalence of the disease which is assumed to be known.

Because ALS is a rare disease with *F *= 0.0001, we can estimate *q*_*i *_from the sampled controls. Thus, we assume that *q*_*i *_= (number of *i*^*th *^genotype in controls)/*m *is known in the following discussion. In the Dom∩Dom model, the 5^*th*^, 6^*th*^, 8^*th *^and 9^*th *^genotype combination {(aA, bB), (AA, bB), (aA, BB), (AA, BB)} is the high-risk genotype combination, and the combination of the other genotypes is the low-risk genotype combination. Let *q*_*H *_= *q*_5 _+ *q*_6 _+ *q*_8 _+ *q*_9 _denote the population frequency of the high-risk genotype combination. Then, the penetrance *α *and *β *(see Figure 1) can be calculated by(1)

Thus, we have only one unknown parameter *R *Let *S *indicate that the two-locus combination of interest shows significant association. As described in the previous section, we use a two-step approach for the two-locus analysis. A significant association of the two-locus combination from our two-step method means that each of the two loci shows significant marginal association at level *α*_1 _in step 1 and significant joint association at level *α*_2 _in step 2. We calculate the likelihood *L*(*R*) using the equation

where the data *D *= {*n*_1_,..., *n*_9_; *m*_1_,..., *m*_9_}. Since the data D constitutes, by definition, a significant result, so D implies S; hence Pr(*S|D,R*) = 1. If the value of *L*(*R*) can be calculated for each given *R*, we can obtain the MLE of *R *by using a numerical optimization method (grid search was used in this article). For each *R*, the numerator can be calculated by the product of two multinomial distributions

where  if the *k*^*th *^genotype is a low-risk genotype;  otherwise. The traditional method to estimate the relative risk is to maximize Pr(*D*|*R*), the numerator in the likelihood function *L*(*R*), without considering the fact that the loci were significant for association. There is no simple method to calculate the denominator Pr(*S|R*), the power of our two-step test. We propose to use a simulation method as described below. For a given *R*, the values of *α *and *β *can be calculated by equation (1). When *α, β*, and *q*_*i *_are known, we can generate the two-locus genotypes for *n *cases and *m *controls. Next, we will perform the single-marker test and the two-locus test on the data set. If the p-values of the two single-marker tests are less than *α*_1 _and the p-value of the two-locus test is less than *α*_2_, the data set is said to be significant for association. We repeat the process to generate the data sets many times (1 million was used in this article). The proportion of significant data sets is the estimate of Pr(*S*|*R*).

When the relative risk *R *has been estimated, the corresponding estimates of *α *and *β *can be obtained from equation (1). The estimate of odds ratio of the high-risk genotype group is given by .

Following Zollner and Prichard, when there are more than two genotype groups in the models such as these in Figure 2, we define the odds ratio of one group to be the odds of this group divided by the odds of the combination of the others. For example, there are three genotype groups in the Dom × Dom model: low risk genotype group *G*_*L *_= {aabb}, middle risk genotype group *G*_*M *_= {aabB, aaBB, aAbb, AAbb}, and high risk genotype group *G*_*H *_= {aAbB, aABB, AAbB, AABB}. The odd ratio of the high risk group *OR*^*H *^is the odds of *G*_*H *_divided by the odds of *G*_*M *_∪ *G*_*L *_= {aabb, aabB, aaBB, aAbb, AAbb}. The odd ratio of the low risk genotype group *OR*^*L *^is the odds of *G*_*L *_divided by the odds of *G*_*M *_∪ *G*_*H *_= {aabB, aaBB, aAbb, AAbb, aAbB, aABB, AAbB, AABB}. The odds ratio estimation method will be the same as the case of two genotype groups.

We used this new proposed method to estimate the odds ratio for each of the two-locus combinations that showed significant association with ALS in our two-locus analysis. Based on the estimated penetrance, we used a simulation method to estimate the sample size required to replicate the findings with 80% power.

## Results

We applied the two-locus analysis with two steps to the genome-wide association data set for sporadic ALS. The analysis was done for all genotypes with a call rate greater than or equal to 95% (549,062 SNPs left). SNPs on the sex chromosome were excluded in the analysis. In the first step, we returned 1,000 SNPs with the smallest p-values which corresponded to use a p-value cut-off *α*_1 _= 0.0023. Then we tested all of the *L *= 499,500 two-locus combinations under each of the seventeen models and used 1,000 permutations to evaluate the adjusted p-value for each of the two-locus combinations. After adjusting for multiple tests and multiple SNPs, we found two two-locus combinations with p-values less than 0.05. There were three SNPs involved in the two two-locus combinations. The details of the three SNPs are given in Table 1. The combination of SNP1 and SNP2 followed the Dom∩Dom model with a p-value of 0.032 and SNP1 and SNP3 followed the Dom × Dom model with a p-value of 0.042. Table 2 gives the number of cases and controls in each of the nine genotypes for the two two-locus combinations. This table shows that the two two-locus combinations fit the two models, Dom∩Dom and Dom × Dom. For example, for SNP1 and SNP2, there were more cases than controls for genotypes with at least one C allele at SNP1 and at least one G allele at SNP2 and there were more controls than cases for the other genotypes, which indicated that SNP1 and SNP2 followed the Dom∩Dom model. In Schymick et al.'s 2 df single-gene analysis [1], SNP1 was ranked 1^st ^with a p-value of 6.8 × 10^-7^, SNP 2 was ranked 10^th ^with a p-value of 2.2 × 10^-5^, and SNP 3 was ranked 2^nd ^with a p-value of 1.7 × 10^-6^.

**Table 1:** Information of the three SNPs. HRA: high-risk allele.

					Allele frequency		
						
SNP	dbSNP ID	Chromosome Location	Gene	Two alleles	Controls	Cases	HRA
				T	0.656	0.505	
					
SNP1	rs4363506	10q26.13	Intergenic	C	0.344	0.495	C

				A	0.467	0.341	
					
SNP2	rs3733242	4q21.1	SHROOM3	G	0.533	0.659	G

				C	0.887	0.786	
					
SNP3	rs16984239	2p24	Intergenic	A	0.113	0.214	A

**Table 2:** (number of cases)/(number of controls) in each of the two-locus genotypes.

		SNP1		
		
SNP	Genotype	TT	TC	CC
SNP2	AA	11/23	14/37	3/7
	
	AG	29/50	73/56	29/11
	
	GG	23/45	65/24	28/16

SNP3	CC	33/95	95/89	37/30
	
	CA	29/20	52/25	22/4
	
	AA	1/3	5/3	1/0

To estimate the impact of the two two-locus combinations on sporadic ALS, we first estimated the penetrance of the two-locus genotypes for each of the two two-locus combinations under the corresponding model. Based on the estimated penetrance, we estimated the relative risk, odds ratio and sample size required to replicate the significant findings with 80% power. We followed what is in Zollner and Pritchard to obtain the 95% CI of the estimates [32], that is, we generated 95% CI by comparing the likelihood of all initial parameter points with the likelihood of the point estimate. We included all points for which twice the difference of log-likelihoods was < 95th percentile of a *χ*^2 ^distribution with 1 df. The estimations using both the proposed method (adjusted estimates) and the traditional method (unadjusted estimates) are summarized in Table 3. From this table, we can see that the unadjusted relative risk, odds ratio were higher than the adjusted ones, and the unadjusted sample size was smaller than the adjusted one. These results were consistent with the finding of others that the traditional estimates of relative risk and odds ratio are up-biased [33,34].

**Table 3:** Penetrence, relative risk and odds ratio of the two-locus combinations.

Two-locus combination		SNP1 and SNP2	SNP1 and SNP3
Penetrance	Unadjusted	*Pen*() = 0.48*F*,	*Pen*() = 0.40*F*;
		*Pen*() = 1.78*F*.	*Pen*() = 1.02*F*;
			*Pen*() = 2.60*F*.
	
	Adjusted	*Pen*() = 0.51*F*;	*Pen*() = 0.44*F*;
		*Pen*() = 1.73*F*.	*Pen*() = 1.03*F*;
			*Pen*() = 2.43*F*.

R and 95% CI	Unadjusted	3.70, (2.85, 4.85)	2.55, (2.10, 3.15)
	
	Adjusted	3.40, (2.40, 4.60)	2.35, (1.85, 2.95)

OR^H ^and 95% CI	Unadjusted	3.70, (2.85, 4.85)	3.37, (2.66, 4.34)
	
	Adjusted	3.40, (2.40, 4.60)	3.05, (2.27, 4.01)

OR^L ^and 95% CI	Unadjusted	0.27, (0.21,0.35)	0.31, (0.23, 0.40)
	
	Adjusted	0.29, (0.22, 0.42)	0.34, (0.25, 0.47)

SS and 95% CI	Unadjusted	680, (480, 1040)	680, (460, 1040)
	
	Adjusted	800, (500, 1500)	810, (520, 1520)

Note: There were two genotype combinations for SNP1 and SNP2,  = {TTAA, TCAA, CCAA, TTAG, TTGG} and  = {TCAG, CCAG, TCGG, CCGG}, three genotype combinations for SNP1 and SNP3,  = {TTCC},  = {TCCC, CCCC, TTCA, TTAA} and  = {TCCA, CCCA, TCAA, CCAA}. *Pen*(*G*) denotes the penetrance of G. R: relative risk. For SNP 1 and SNP2, *R *= *pen*()/*pen*() = *α/β*; for SNP1 and SNP3, *R *= *pen*()/*pen() = pen*()/*pen*() = *θ*. OR^H ^(OR^L^): the odds ratio of the high-risk (low-risk) genotype group. SS: the sample size required to reach 80% power. Adjusted (Unadjusted): based on the penetrance estimated using the method proposed in this article (the traditional method). *F *is the prevalence and *F *= 10^-4^.

## Discussion

In this study we proposed a new analytical method that considered joint effects of genes to analyze a data set from the GWAS in sporadic ALS previously performed by Schymick et al. [1]. Our analysis showed that the combination of SNP1 and SNP2 and the combination of SNP1 and SNP3 had significant effects on sporadic ALS.

Population stratification may lead to false-positive results. We had also checked the population stratification problem in this data set using the following method. We randomly chose 5,000 SNPs and got their p-values by a single marker test. If population stratification did exist in this data set, among the 5,000 p-values, there should be more small p-values than expected under the uniform distribution. We used the one-side Kolmorgorov test statistic to test if the 5,000 p-values followed a uniform distribution. We repeated the procedure 10 times. The Kolmorgorov test results showed that the p-values followed a uniform distribution for all 10 replications, which indicated that there was no population stratification in this data set. The lack of population stratification in the data set was consistent with the results of Schymick et al. [1]. Schymick et al. studied the potential population structure in this data by using STRUCTURE program [38]. The analysis with STRUCTURE showed that there was no discernible difference in the population substructure between cases and controls.

Significant associations claimed by association studies often fail to be replicated. One possible reason is the overestimation of the effect in terms of the odds ratio or relative risk of the claimed variants. The overestimation of the effect leads to the underestimation of the sample size required to replicate the finding. In this article, we proposed a new method to estimate the effect of claimed variants. Based on the study of Zollner and Pritchard [32], we expected that the estimates of odds ratio and relative risk based on our proposed method would be nearly unbiased. Thus we provided a useful tool to estimate the sample size for the follow up studies. For example, in order to replicate the finding of SNP1 and SNP2 (the adjusted p-value less than 0.05 under the Dom ∩ Dom model) with 80% power, the sample size required is 800 estimated using our proposed method instead of 680 estimated using the traditional method.

Currently, several methods are available to test associations by taking joint effects of genes into account, such as combinatorial searching method (CSM) and the multifactor dimensionality reduction (MDR) method [39,40]. We used the two-step CSM and MDR, replacing the two-locus analysis test in step 2 by the CSM or MDR, to perform the two-locus analysis. For the two-step MDR, we returned 50 SNPs instead of 1, 000 SNPs in the first step due to the computational intensity. Both of the two-step CSM and MDR found rs4363506 (SNP1) and rs12680546 (on chromosome 8) as the best two-locus combination. However, the adjusted p-values of the two-step CSM and MDR were 0.2 and 0.156. This means that the two-step CSM and MDR did not find any two-locus combinations that had significant association with sporadic ALS. The possible reasons are as follows: The genotypes of the two-locus combinations we found (such as those given in Table 3) are ordered. For example, penetrance of *H*_1_*H*_2 _≥ penetrance of *H*_1_*h*_2 _≥ penetrance of *h*_1_*h*_2_, where *H*_1_(*h*_1_) and *H*_2_(*h*_2_) are the high-risk (low-risk) genotypes in the first and second marker, respectively. The CSM and MDR ignore the order of genotypes and therefore can group any two genotypes together-in essence searching for the "best" one among 21,146 different partitions of the two-locus genotypes. By searching for irrelevant two-locus genotype combinations, the CSM and MDR did not gain more information but increased the noise level, and thus lost power.

## Conclusion

The proposed two-stage analytical method can be used to search for two-locus joint effects of genes in GWAS. The two-stage strategy significantly decreased the computational time and the multiple testing burdens associated with GWAS. We have also observed that the three SNPs identified by our two-stage strategy can not be detected by single-locus tests.

## Competing interests

The authors declare that they have no competing interests.

## Authors' contributions

QS and SZ designed the study. ZZ contributed the two-locus data analysis under the direction of SZ. SZ performed the penetrance estimation. JCS & BJT assisted in data interpretation and approved the final manuscript. QS and SZ contributed to the writing of the manuscript. All authors read and approved the final manuscript.

## Pre-publication history

The pre-publication history for this paper can be accessed here:

http://www.biomedcentral.com/1471-2350/10/86/prepub
